# High-risk factors associated with refractory childhood bacterial meningitis in Southwest China

**DOI:** 10.1186/s12887-023-04007-z

**Published:** 2023-05-05

**Authors:** JinFeng Wu, XiaoJie Song, Yue Hu, Jin Chen, Li Jiang

**Affiliations:** 1 Department of Neurology, Children’s Hospital of Chongqing Medical University, Chongqing, China; 2 National Clinical Research Center for Child Health and Disorders, Chongqing, China; 3 Ministry of Education Key Laboratory of Child Development and Disorders, Chongqing, China; 4 China International Science and Technology Cooperation Base of Child Development and Critical Disorders, Chongqing, China; 5 Chongqing Key Laboratory of Pediatrics, Chongqing, China

**Keywords:** Childhood, Refractory bacterial meningitis, High-risk factors

## Abstract

**Background:**

Refractory bacterial meningitis is acute, develops rapidly, and has higher mortality and morbidity than common bacterial meningitis. This study was undertaken to investigate the high-risk factors related to refractory bacterial meningitis in children with positive pathogens.

**Methods:**

We retrospectively analyzed the clinical data of 109 patients who had bacterial meningitis. The patients were divided into a refractory group (96 patients) and nonrefractory group (13 patients) based on the classification criteria. Seventeen clinical variables on risk factors were extracted and evaluated by univariate and multivariate logistic regression analyses.

**Results:**

There were 64 males and 45 females. The onset age ranged from 1 month old to 12 years old, and the median age was 181 days old. The pathogenic bacteria included 67 cases of gram-positive (G+) bacteria (61.5%) and 42 cases of gram-negative (G-) bacteria. In patients who were 1 to 3 months old, *E. coli* was the most common (47.5%), followed by *Streptococcus agalactiae* and *Staphylococcus hemolyticus* (10.0%); in patients > 3 months old, *S. pneumoniae* was the most common (55.1%), followed by *E. coli* (8.7%). The multivariate analysis indicated that consciousness disorder (odds ratio [OR] = 13.050), peripheral blood C-reactive protein (CRP) ≥ 50 mg/L (OR = 29.436), and the isolate bacteria being gram-positive bacteria (OR = 8.227) were independent risk factors for predicting who would progress to refractory bacterial meningitis in this group.

**Conclusion:**

For patients who have pathogenic positive bacterial meningitis along with consciousness disorder, CRP ≥ 50 mg/L, and/or have an isolate bacteria that is a G + bacteria, it is important to be alert to the potential for progression to refractory bacterial meningitis, which demands the physicians’ significant attention.

## Background

Bacterial meningitis is an inflammation of the meninges caused by various pathogens, and some lesions can affect the brain parenchyma. It is a common central nervous system infectious disease in children. The main clinical features of the disease include fever, increased intracranial pressure, meningeal irritation, and purulent changes to the cerebrospinal fluid (CSF) [1]. Even with effective anti-infection treatment, 30-50% of survivors have permanent neurological sequelae[2], and the most common early neurological complication is subdural effusion[3]. Approximately 10% of children may have long-term neurological sequelae, including epilepsy, neuropsychological disorders and hearing impairment[4]. In the past few decades, the pathogenic bacteria categories of bacterial meningitis have undergone tremendous changes[5], which are closely related to the patient’s age, location, and autoimmune status [6]. In Europe and America, the most common pathogenic bacteria in children are S. pneumoniae, N. meningitidis and *Escherichia coli*[7, 8]. In China, the more common pathogenic bacteria are *Streptococcus pneumoniae*, *Escherichia coli* and Group B hemolytic streptococcus.

Clinically speaking, some pediatric patients have poor therapeutic outcomes, such as minimal effectiveness of conventional antibiotic therapy, rapid relapse after showing signs of improvement treatment, persistent abnormal CSF and peripheral blood inflammation indicators, and serious sequelae, including death. They are diagnosed with refractory bacterial meningitis. The onset of refractory bacterial meningitis is acute, it develops rapidly, and mortality and morbidity are higher than in cases of common bacterial meningitis[9]. According to reports, 37.5-50.9% of bacterial meningitis cases will be refractory[9, 10]. Refractory bacterial meningitis is difficult to treat, expensive to treat, and poor in prognosis and has always been the focus of attention in the Department of Pediatric Neurology and Infection. Early diagnosis and timely and rational antibiotic treatment are the keys to improving prognosis.

The current detection rate of pathogens is low, at approximately 13.8–33.6% in China[11, 12]. We believe that the lack of evidence of pathogenic bacteria leading to the inability to give appropriate antibiotics based on antimicrobial susceptibility testing (AST) may be the most important reason for refractory bacterial meningitis. In our center, we do not yet understand why many patients with bacterial meningitis with an established pathogen have a poor response, prolonged treatment, and a poor prognosis even with antibiotics based on AST. Therefore, this study selected children with etiologically positive bacterial meningitis as the research subjects, aiming to identify the high-risk factors for progression to refractory bacterial meningitis in this group, in order to provide clinical basis for early diagnosis and treatment of refractory suppurative meningitis.

## Methods

### Subject inclusion and exclusion criteria

For inclusion in the study, all subjects had to meet the following criteria: (i) Symptoms and findings consistent with the diagnostic criteria for bacterial meningitis [13, 14]: ① Acute or subacute onset, high fever, headache, vomiting, varying degrees of disturbance of consciousness, convulsions, with young infants having irritability, restlessness, and staring. ② The patient has positive symptoms of meningeal irritation and increased intracranial pressure; young infants may show full anterior fontanel and widened cranial sutures. ③ Cerebrospinal fluid examination showed an increase in the number of nucleated cells (mainly neutrophils), an increase in protein content, and a decrease in glucose content. (ii) Only one pathogen was cultured in the CSF and/or peripheral blood.

Subjects would be excluded meet the following criteria: (i) Combined with viral encephalitis, tubercular meningitis, fungus meningitis, cryptococcal meningitis, or encephalopathy caused by noninfectious factors. (ii) Patients with incomplete data were not included.

Pediatric patients meeting one or more of the following criteria were classified as having refractory bacterial meningitis[9, 10, 15–19]: (i) the duration of antibiotics based on empirical or drug sensitive tests had exceeded the standard time of > 14 days for G + bacteria and > 21 days for G − bacteria, and the clinical manifestations, peripheral blood inflammation indicators, and/or CSF indicators were still abnormal; (ii) complications, such as subdural effusion, ependymitis, and hydrocephalus; (iii) death or sequelae such as secondary epilepsy, cranial nerve damage, and[/or] psychomotor retardation occurred during late follow-up; and (iv) the existence of unexplained recurrent suppurative intracranial infections, with recurrent meningitis defined as at least two episodes of headache, fever, and meningismus with associated cerebrospinal fluid (CSF) pleocytosis, separated by a period of full recovery[20].

### Clinical information

the patients were admitted to Children’s Hospital of Chongqing Medical University (the largest children’s medical center in southwest China) between January 1,2014 and September 1, 2018. There were at least a 3-year follow-up before September 1, 2021.

The following clinical information was collected: (i) general information: sex, age, and length of hospital stay. (ii) Clinical manifestations at admission: duration of fever, mental fatigue, convulsions, vomiting, disturbance of consciousness (including somnolence, stupor, coma), slow/loss of pupillary light reflex, unequal pupils, meningeal irritation, and Babinski sign. (iii) Initial auxiliary examination: peripheral blood: white blood cell counts (WBC 4.0–12.0*10^9^/L), platelets (PLT 100–380*10^9^/L), hemoglobin (HB 110-150 g/L), C-reactive protein (CRP < 8 mg/L), and procalcitonin (PCT < 0.1 ng/dL); CSF: WBC counts (< 15*10^6^/L), protein value(0.15–0.45 g/L ), and glucose value (2.44–4.44 mmol/L). (iv) Bacterial culture and AST were performed using an automated instrument method (Vitek Compact, France). Interpretation of AST results was carried out in accordance with the standards of Clinical and Laboratory Standards (CLSI). Drug Resistant bacteria The identification of *Staphylococcus epidermidis must* be two positive blood culture results at different sites or times or both blood and cerebrospinal fluid cultures that are positive. (v) Causes/other factors include respiratory tract infection, digestive tract infection, ear infection, congenital nervous system defect, head trauma, etc. (vi) Complications included subdural effusion/pus, secondary epilepsy, hydrocephalus, ependymitis, cerebral hernia, cranial nerve injury, hemiplegia, hearing impairment, encephalomalacia, and brain parenchyma changes (cranial imaging). (vii) Follow-up: telephone counseling about the recovery of the children’s family members after discharge and whether they have sequelae, including deafness, secondary epilepsy, movement disorders, hydrocephalus, psychomotor retardation, etc.

### Data analysis

Statistical analysis was performed using the SPSS22.0 statistical software package. Descriptive analyses of the count and measurement data were performed using proportions, frequencies, medians, ranges, means, and SD. The chi-square test or Fisher’s exact test was used to compare count data. Measurement data were compared using the rank sum test. To identify high-risk factors related to refractory bacterial meningitis, multivariate logistic regression analysis was used for the indicators with statistical significance in the univariate statistical analysis. Statistical significance was established at P < 0.05.

## Results

### General clinical characteristics

The study included 109 patients with bacterial meningitis who met the above inclusion and exclusion criteria. There were 64 males and 45 females. The onset age ranged from 1 month old to 12 years old, and the median age was 181 days old. The median total duration of the hospital stay was 24 days (2–115 days). The patient who was hospitalized for 2 days was discharged from the hospital because his family members gave up on treatment, and the patient died after discharge.

In this group, 82 patients (75.2%) had precursor infection foci, which was followed in number by pneumonia in 66 patients (60.6%), digestive tract infection in 31 patients (28.4%), ear and nose inflammation in 16 patients (14.7%), skin tissue suppurative infection in 21 patients (1.8%), and urinary tract infection in 2 patients (1.8%).

Nineteen patients (17.4%) had one or more other factors, including 4 patients with both cerebrospinal fluid leakage and traumatic brain injury, 4 patients only with cerebrospinal fluid leakage, 4 patients only with traumatic brain injury, 6 patients with immune insufficiency or deficiency (5.5%), 1 patient (0.9%) with previous medical history because of Mondini deformity of the inner ear. Among the patients, 5 patients had underlying diseases, including 4 patients with congenital heart disease, 1 patient with a urinary tract malformation, and 1 patient with hemophagocytic syndrome.

All children underwent blood analysis on admission, of which 64 patients (58.7%) had abnormal WBC counts (< 4.0*10^9^/L or > 12.0*10^9^/L), 67 patients (61.5%) had abnormal PLT counts (< 100* 10^9^/L or > 600*10^9^/L), and 25 patients (22.9%) had moderate to severe anemia (HB < 90 g/L). Ninety-two patients (84.4%) had elevated CRP values (≥ 8 mg/L), of which 79 patients (72.5%) had CRP values ≥ 50 mg/L. A total of 104 patients (95.4%) had increased PCT values (≥ 0.1 ng/dl), of which 22 patients (22.9%) had PCT levels > 30 ng/dl.

A total of 107 patients underwent a cerebrospinal fluid examination on admission (the other 2 patients did not undergo the examination due to suspected brain herniation), of which 45 patients (42.1%) had CSF-WBC count ≥ 500*106/L, 79 patients (73.8%) had CSF protein > 1.0 g/L, and 41 patients (38.3%) had CSF glucose < 1.50 mmol/L.

### Etiology

Blood cultures and/or CSF cultures were tested for all 109 patients; 86 patients (78.9%) had positive blood culture findings, and 47 patients (43.1%) had positive CSF culture findings, 24 patients had both positive CSF culture findings and blood culture findings. Among the pathogenic bacteria, G + bacteria (61.5%) were more common than G − bacteria. The most common G + bacteria were *Streptococcus pneumoniae* (35.8%), and the most common G − bacteria were *Escherichia coli* (22.9%).

We analyzed the correlation between the patient’s age and pathogenic bacteria (Table 1). In the patients 1 to 3 months old, *E. coli* was the most common (47.5%), followed by *Streptococcus agalactiae* and *Staphylococcus hemolyticus* (10.0%); in the patients > 3 months old, *S. pneumoniae* was the most common (55.1%), followed by *E. coli* (8.7%).

**Table 1:** Etiology of bacterial meningitis in children of different ages (N = 109)

Isolated bacteria	1–3 months (n = 40)	> 3 months (n = 69)	Total (N = 109)
G + bacteria	15(37.5%)	52(75.4%)	67(61.5%)
*Streptococcus pneumoniae*	1(2.5%)	38(55.1%)	39(35.8%)
*Streptococcus agalactiae*	4(10.0%)	1(1.5%)	5(4.6%)
*Staphylococcus epidermidis*	3(7.5%)	2(2.9%)	5(4.6%)
*Staphylococcus hominis*	1(2.5%)	4(5.8%)	5(4.6%)
*Staphylococcus haemolyticus*	4(10.0%)	0(0%)	4(3.7%)
Other G + bacteria^a^	2(5.0%)	7(10.1%)	9(8.2%)
G − bacteria	25(62.5%)	17(24.6%)	42(38.5%)
*Escherichia coli*	19(47.5%)	6(8.7%)	25(22.9%)
*Pseudomonas aeruginosa*	0(0%)	4(5.8%)	4(3.7%)
Other G − bacteria^b^	6(15.0%)	7(10.1%)	13(11.9%)

^a^Other G + bacteria (1–3 months): *Enterococcus* 2; (> 3 months): *Listeria monocytogenes* 2, *Enterococcus* 1, *Staphylococcus warneri* 1, *Staphylococcus capitis* 1, *Streptococcus pyogenes* 1, *Lysinibacillus sphaerucus* 1. ^b^Other G − bacteria (1–3 months): *Neisseria meningitidis* 2, *Klebsiella pneumoniae* 1, *Acinetobacter baumanii* 1, *Enterobacter cloacae* 1, *Stenotrophomonas maltophilia* 1; (> 3 months): *Neisseria meningitidis*1, *Klebsiella pneumoniae* 1, *Acinetobacter baumanii* 1, *Salmonella typhimurium* 1, *Haemophilus influenzae* 1, *Pseudomonas putida* 1, *Brucella melitensis* 1.(the numbers that follow the bacterial names means the number of cases in which that pathogen was identified)

We quantified the results of AST of the main pathogenic bacteria (those occurring in four or more patients) among this group of patients. Among the main G + pathogens, the resistance rate of *S. pneumoniae* to penicillin was 51.3% and to third-generation cephalosporin was 12.8%, and no strain resistant to vancomycin was found. The resistance rate of *Staphylococcus* to penicillin was 92.9%. There was no resistance to penicillin or cephalosporin antibiotics among *S. agalactiae*. Of the main G − pathogens, the resistance rate of *E. coli* to the third-generation cephalosporin was 56.0%, and all G − bacteria were sensitive to carbapenems. The resistance rate of *Pseudomonas aeruginosa* to the third-generation cephalosporin was 100.0%.

### Treatment and follow-up

Antibiotics were used empirically after admission, and the antibiotics were adjusted according to the clinical treatment response, pathogen culture results, and drug sensitivity tests. Table 2 shows the use of antibiotics in 109 children with positive BM after admission. In 77 patients, the clinical manifestations, peripheral blood inflammation indicators, and/or CSF indicators were still abnormal after a full course of antibiotics. They had received a longer course of anti-infective therapy or combined anti-infective regimens.

**Table 2:** The antibiotic choice of 109 patients during hospitalization

Antibiotic Therapy	N
Third-generation cephalosporin	9
Chloramphenicol	14
Penicillin + Chloramphenicol	6
Third-generation cephalosporin + Penicillin	3
Third-generation cephalosporin + Vancomycin/Linezolid	22
Third-generation cephalosporin + Chloramphenicol	2
Vancomycin/Linezolid + Chloramphenicol	38
Vancomycin + Chloramphenicol + Rifampicin	7
Vancomycin + Chloramphenicol + Levofloxacin	2
Vancomycin + Chloramphenicol + Amikacin	2
Vancomycin + Chloramphenicol + Gentamicin	1
Vancomycin + Chloramphenicol + Teicoplanin	1
Third-generation cephalosporin + Rifampicin/Sulfamethoxazole	1
Chloramphenicol + Levofloxacin	1

Seventy-three patients had complications during hospitalization (66.9%), of which 21 cases had two complications, 8 cases had three complications,5 cases had four complications,1 cases had six complications,1 cases had seven complications. 44 patients had subdural effusion (40.4%), 33 patients had brain parenchyma changes (30.3%), 27 patients had hydrocephalus (24.8%), 8 patients had cranial nerve injury (7.3%), 6 patients had hearing loss (5.5%), 6 patients had secondary epilepsy (5.5%), 6 patients had cerebral hernia (5.5%), 3 patients had ventriculitis (2.8%), and 3 patients had hemiplegia (2.8%). One patient had a recurrent condition because of Mondini inner ear malformation (0.9%).

At the final follow-up, the mortality rate was 18.3% in our study (20/109). For the 89 surviving patients, follow-up after discharge from the hospital ranged from 2 year to 7 year 8 m, with 42 patients with retarded psychomotor development (47.2%), 15 patients with cerebral atrophy (13.8%), 11 patients with hearing impairment (10.1%), 10 patients with hydrocephalus (9.2%), 5 patients with cranial nerve injury (4.6%), 4 patients with secondary epilepsy (3.7%), and 3 patients with hemiplegia (2.8%).

In summary, a total of 96 patients (88.1%) were included in the refractory group, the remaining 13 patients (11.9%) received a full course of antibiotics, the clinical symptoms improved, and the indicators of peripheral blood and cerebrospinal fluid returned to normal. No complications occurred, and no sequelae occurred during follow-up. These patients were included in the nonrefractory group.

### The analysis of high-risk factors associated with refractory bacterial meningitis

Based on clinical experience and research, we selected some common clinical indicators for univariate analysis to obtain refractory-related factors. Refractory bacterial meningitis was associated with the following eight clinical indicators (Table 3): disturbance of consciousness, slow pupillary light reflex, CRP ≥ 50 mg/L upon admission, procalcitonin > 30 ng/dL upon admission, CSF WBC count ≥ 500 × 10^6^ cells/L, CSF protein values > 1.0 g/L, CSF glucose content < 1.5 mmol/L, and the isolate bacteria being G + bacteria.

**Table 3:** The Univariate analysis for factors related to childhood refractory bacterial meningitis

Clinical Factor	Refractory group (n = 96)	Non-refractory group (n = 13)	P-value
Gender (male)	57(59.4%)	7(53.8%)	0.704
Age ≤ 1 year	59(61.5%)	10(76.9%)	0.278
Duration of fever ≥ 7 days	61 (63.5%)	6 (46.2%)	0.227
mental fatigue	84 (87.5%)	9 (69.2%)	0.184
Convulsion	44 (45.8%)	4 (30.8%)	0.466
Vomiting	58 (60.4%)	9 (69.2%)	0.757
Disturbance of consciousness	55 (57.3%)	1 (7.7%)	0.002*
Unequal pupil size	12 (12.5%)	0 (0%)	0.353
Slow/loss of pupillary light reflex	42 (43.8%)	1 (7.7%)	0.028*
Positive meningeal irritation	53 (55.2%)	4 (30.8%)	0.174
Positive Babinski sign	45 (46.9%)	5 (38.5%)	0.783
CRP(≥50 mg/L)	77 (80.2%)	2 (15.4%)	< 0.001*
Procalcitonin (>30 ng/dL)	25 (26.0%)	0 (0%)	0.037*
CSF WBC(≥500 × 10^6^/L)	46 (47.9%)	1 (7.7%)	0.014*
CSF protein (> 1.0 g/L)	76 (79.2%)	5 (38.5%)	0.005*
CSF glucose(<1.5 mmol/L)	40 (41.7%)	1 (7.7%)	0.039*
G + bacteria	64 (66.7%)	3 (23.1%)	0.006*

Then, we performed a multivariate analysis of the above 8 clinical factors identified as predicting refractory bacterial meningitis in the univariate analysis. Three factors remained independent risk factors for predicting refractory bacterial meningitis (Table 4): disturbance of consciousness, CRP ≥ 50 mg/L upon admission, and the isolate bacteria being G + bacteria.

**Table 4:** Multivariate analysis for factors associated with childhood refractory bacterial meningitis

Clinical Factor	P-value	OR	95% CI
Disturbance of consciousness	0.031	13.050	1.270−134.071
CRP(≥50 mg/L)	< 0.001	29.436	4.651−186.301
G + bacteria	0.017	8.227	1.455−46.520

OR, odds ratio; CI, confidence interval

## Discussion

At present, in China, the most common pathogen causing bacterial meningitis in the neonatal period is *E. coli*; the most common pathogens in children with bacterial meningitis at the ages of 1 to 3 months are *E. coli*, Group B hemolytic streptococcus, and *S. pneumoniae*; and *S. pneumoniae* is the most common etiologic agent for bacterial meningitis in children over 3 months of age [21]. This is basically in line with our results: children between 1 and 3 months old were predominantly infected with *E. coli*, followed by *S. agalactiae* and *S. hemolyticus*; children older than 3 months were predominantly infected with *S. pneumoniae*, followed by *E. coli*. We found that the incidence of *S. agalactiae* in bacterial meningitis patients who were 1 to 3 months old was slightly prominent, which is consistent with some recent studies in China that have concluded that the constituent ratio of *S. agalactiae* in meningitis is increasing [22]. In addition, staphylococci were also slightly prominent in patients between 1 and 3 months of age, which may be related to their immune insufficiency,so physicians should pay attention to the conditional pathogens causing infection.

*Escherichia coli* is most common in infants aged 1–3 months, and third-generation cephalosporins and/or meropenem are recommended for the initial treatment. If the patient infected with *Streptococcus agalactiaet*, ampicillin or penicillin G may be the first choice, If the patient infected with *Streptococcus agalactiaet* is seriously ill at the early course, a third-generation cephalosporin and/or vancomycin may be the first choice [21, 23]. *Streptococcus pneumoniae* is the most common infection in children older than 3 months, and third-generation cephalosporins combined with vancomycin are the first choice for anti-infection, If the efficacy is not good, antibiotics should be upgraded in time; rifampicin, aminoglycosides, sulfonamides, or quinolones can also be used in combination [21, 24, 25]. Appropriately extending the course of treatment according to the complications may also be a feasible means to improve the prognosis.

In this group of patients, the refractory rate was as high as 88.1%. This may be related to the selection of research subjects with a positive etiology. Studies have suggested that positive blood culture or cerebrospinal fluid culture is a high-risk factor for poor prognosis in BM [26–28]. This study had ear and nose inflammation (14.7%), and another 19 patients (17.4%) had other disease factors. Some studies believe that ear and nose inflammation, cerebrospinal fluid leakage, traumatic brain injury, inner ear deformity, immune insufficiency or defects increase the risk of refractory BM[20, 28]. There are also studies that suggest that genetic variation can affect the severity and prognosis of BM in addition to differences in immune responses. Ferwerda et al. [29] sequenced the coding region of the innate immune gene in children with *Streptococcus pneumoniae* meningitis and found that CXCL1 and CARD8 mutations are associated with susceptibility and that NOD2 and IRAK4 are associated with pneumococcal meningitis. In addition, our medical center is located in Southwest China, and the low economic level may also be one of the reasons.

Huo et al. [10] suggested that the high-risk factors for refractory bacterial meningitis include a longer duration of fever, higher CRP value, and a higher adenosine deaminase value in the CSF, but the study did not quantify the relevant indicators. Peng et al. [9] found that unequal pupils and CSF glucose < 1.5 mmol/L are high risk factors for poor prognosis of refractory bacterial meningitis, but there is a lack of further analysis of factors associated with pathogenic positive bacterial meningitis. Based on our previous research and clinical experience, we selected 23 factors related to refractory outcome or poor prognosis for statistical analysis [30–32] and found that early-stage consciousness disorder, CRP ≥ 50 mg/L, and the isolate bacteria being G + bacteria were independent risk factors for pathogenic positive bacterial meningitis cases to progress to refractory outcomes.

Disturbance of consciousness results from damage to the ascending reticular activating system and cerebral cortex. Several studies have shown that disturbance of consciousness or coma is an independent risk factor leading to poor prognosis for children with bacterial meningitis [30, 31, 33], and it is also one of the factors for refractory outcomes. Therefore, for bacterial meningitis patients with consciousness disorders, especially coma, it is necessary to pay close attention to whether there are significant increases in intracranial pressure, brain herniation, and/or brainstem dysfunction, as these must be actively treated to yield a better outcome.

CRP is an acute-phase protein and is synthesized by the liver under conditions of stress. It is highly sensitive to inflammation and has become an indicator that is widely used in clinical assistance to diagnose bacterial meningitis. The increase in the CRP value reflects the severity of the infection [30]. Similarly, the CRP value can also help predict the prognosis and outcome [6, 25, 32]. Liu Yuming et al. [34] believed that CRP > 100 mg/L is indicative of a poor prognosis for children with pneumococcal meningitis. Our study found that children with bacterial meningitis whose serum CRP was significantly elevated at the beginning of the disease were more likely to develop refractory bacterial meningitis, especially for those with CRP ≥ 50 mg/L. Therefore, for such cases, we recommend pursuing pathogenic examination as soon as possible and administering aggressive antibiotic treatment.

We found that the isolate bacteria being G + bacteria was one of the factors associated with refractory bacterial meningitis. The main pathogenic G + bacteria identified in our group included *S. pneumoniae*, followed by *S. agalactiae* and *Staphylococcus* species. The mucosa of the upper respiratory tract of infants and young children is rich in blood vessels and lymphoid tissues but lacks cilia and has limited immunological capacity to clear bacteria. *S. pneumoniae* often colonizes these sites and is prone to cause blood-borne infections and spread. Furthermore, *S. pneumoniae* can release bacterial toxins to trigger the body’s inflammatory response, leading to an increase in inflammatory cells and the release of inflammatory mediators in the CSF, resulting in complications such as cerebral ischemia, cerebral edema, hydrocephalus, and increased intracranial pressure [35]. Therefore, *S. pneumoniae* is a major factor in the sequelae and dysfunction of the nervous system in bacterial meningitis and has become an important pathogen associated with refractory outcomes [36, 37]. *S. agalactiae* is a common pathogen of bacterial meningitis in patients younger than 3 months. Some clinical studies indicate that *S. agalactiae* is one of the pathogens involved in refractory bacterial meningitis, primarily because children infected with *S. agalactiae* are prone to complications [19, 38, 39] that lead to a prolonged treatment time. This may be because the *S. agalactiae* bacteria that cause bacterial meningitis are mostly serotype III [40, 41], which can strongly adhere to the vascular endothelial tissue, chorion, and lungs and thus these bacteria are difficult to remove and treat.

Among this patient population, the resistance rate to penicillin was 51.3% for *S. pneumoniae* and 92.9% for *Staphylococcus*. The drug resistance rate was higher in the refractory group, so the drug resistance of the pathogenic bacteria may be related to the difficulty in treatment.

There are several limitations of this study. First, its retrospective nature has the inherent weakness of precluding evaluation of the suitability of current practices in the management of bacterial meningitis. Second, the patients were from only a single clinic center, so the low number of patients in our study might influence the statistical significance of the results.Third, the lack of analysis of bacterial resistance may make the results less comprehensive.

## Conclusions

Based on age and drug resistance in the region, empiric treatment should be given to suspected patients as soon as possible. Early pathogenic examination and anti-infective treatment based on a drug susceptibility test for the isolated pathogens are strategies for avoiding refractory disease and a poor prognosis. Moreover, doctors should not let their guard down, especially for patients with consciousness disorders, CRP ≥ 50 mg/L, and/or isolated G + bacteria, because they are very likely to progress to refractory bacterial meningitis.Appropriate extension of anti-infective course, selection of rational combined anti-infective regimen, active treatment of complications and discovery of underlying etiology may be helpful to improve the prognosis of refractory bacterial meningitis.

## Data Availability

All data generated or analysed during this study are included in this published article and its supplementary information files.

## Abbreviations

AST: antimicrobial susceptibility testing
CI: confidence interval
CRP: C-reactive protein
CSF: cerebrospinal fluid
G- bacteria: Gram-negative bacteria
G + bacteria: Gram-positive bacteria
OR: odds ratio
PLT: platelets
HB: hemoglobin
PCT: procalcitonin
WBC: white blood cell.
